# Evaluating the presence of IAN injury in patients with juxta-apical radiolucency after third molar surgery: a retrospective cohort study

**DOI:** 10.1186/s12903-021-01785-9

**Published:** 2021-09-05

**Authors:** Mahvash Hasani, Nasim Razavi, Abdolaziz Haghnegahdar, Motahhareh Zarifi

**Affiliations:** 1 Department of Oral and Maxillofacial Radiology, School of Dentistry, Shiraz University of Medical Sciences, Shiraz, Iran; 2 School of Dentistry, Shiraz University of Medical Sciences, Shiraz, Iran

**Keywords:** Juxta-apical radiolucency, IAN injury, Inferior alveolar nerve, Cone-beam computed tomography

## Abstract

**Background:**

Juxta-apical radiolucency (JAR) has been presented as a radiographic sign, suggestive of the IAN injury through third molar surgery. This study aimed to evaluate the relation of JAR with IAN injury in cone-beam computed tomography (CBCT) images and to determine whether the presence of JAR is related to tooth angulation, proximity to the mandibular canal, position to the IAN, and thinning of the cortical plates.

**Methods:**

Of an initial sample of 545 mandibular third molars, a total of 75 JAR^**+**^ and 75 JAR^−^ teeth were evaluated by CBCT. We assessed the relationship between the presence of JAR in cone-beam computed tomography (CBCT) images and the presence of IAN injury after mandibular third molar surgeries. Moreover, we investigated whether the presence of IAN injury is related to tooth angulation, proximity to the mandibular canal, position to the IAN, and thinning of the cortical plates. Descriptive statistics, Chi-square test, and Fisher’s exact test were performed for statistical analysis.

**Results:**

A significant relationship was found between JAR and temporary IAN injury (*P* = 0.036). However, there was no case of permanent IAN injury. IAN injury showed no significant relationship with the tooth angulation, position to IAN and proximity to the mandibular canal, lingual cortical plate thinning, sex, and age.

**Conclusions:**

JAR is generally in contact with the mandibular canal, and some degree of cortical thinning can be found in most cases. In this study, JAR was significantly related to temporary IAN injury. JAR may increase the risk of nerve injury during the surgical removal of third molars.

## Background

The mandibular third molars are the most commonly impacted teeth [1]. Extraction of impacted or erupted mandibular third molars is one of the most frequent dentoalveolar surgical procedures [2]. These teeth are in close proximity to important structures, including the inferior alveolar nerve, the lingual nerve, and the adjacent second molars. Generally, injury to the inferior alveolar nerve is a major concern for surgeons. The incidence of temporary and permanent inferior alveolar nerve neuropathy, associated with mandibular third molar surgeries, is estimated at 1–20 % and 0-3.6 %, respectively [3, 4]. The majority of injuries result in transient sensory disturbances, while in some cases, permanent paresthesia, hypoesthesia, or even dysesthesia may occur. These sensory disturbances can cause speech and mastication problems and adversely affect the patient’s quality of life. They also constitute one of the most frequent causes of medical complaints and litigation [5].

The radiographic position of the mandibular third molars relative to the mandibular canal has been shown to be useful in assessing the risk of damage to the inferior alveolar nerve following extraction [6–8]. Seven radiographic signs can increase the incidence of injury to the inferior alveolar nerve, including the darkening of the roots, diversion of the canal, and interruption of the white line of the canal, which are assumed to be the best predictive signs of neurosensory deficits [9]. In 2005, Renton et al. introduced the juxta-apical area (JAA) or juxta-apical radiolucency (JAR) as a new predictive sign [4]. JAR is a well-defined area of radiolucency that is apical or lateral to the roots of mandibular third molars.

In 2010, Umar et al. showed that JAR is a large cancellous bone space, which is superimposed on the inferior alveolar canal and is not always in contact with it [10]. Other researchers have also investigated the relationship between JAR, the inferior alveolar nerve (IAN), and the third molar roots [11–13]. They established that JAR is commonly located buccally or superiorly to the canal and introduced the concept of a JAR-associated IAN injury. They suggested that thinning of the cortical plate could be responsible for postoperative IAN injury following the extraction of third molars [12, 14]. Moreover, Gilvetti et al. evaluated IAN injury in patients with JAR following the third molar surgery, based on panoramic radiography. According to their study, the presence of JAR is not a reliable predictor of the risk of permanent injury to the inferior alveolar nerve [9].

There are some controversies regarding the nature of the JAR as a risk factor for inferior alveolar injuries. Considering the limitations of previous studies, which mostly applied panoramic radiography [9, 13] and lacked a control group [9, 15], the goal of this study was to determine the relationship between the presence of JAR in cone-beam computed tomography (CBCT) images and the presence of IAN injury after mandibular third molar surgeries in the JAR^+^ and JAR^−^ groups. Furthermore, we scrutinized whether the presence of JAR is related to tooth angulation, proximity to the mandibular canal, position to the IAN, and thinning of the cortical plates.

## Methods

This retrospective cohort study was approved by the ethics committee of Shiraz University of Medical Sciences, Shiraz, Iran (#IR.SUMS.DENTAl.REC.1399.041). The study population consisted of all patients, who underwent lower third molar removal from January 2019 to February 2020, with CBCT images available in the Dental School database of Shiraz University of Medical Sciences. CBCTs were prescribed for all people, whose third molars were closely related to the IAN canal in panoramic views. We divided the image archive into two groups with and without JAR. For both JAR + and JAR- groups, all features, including inclusion and exclusion criteria, are taken into account. This study comprised CBCT images of patients, who had at least one fully impacted lower third molar and were at least 18 years old at the time of surgery. On the other hand, CBCT images of patients were excluded if their lower third molars were associated with active caries lesions extending into the dentine; large restorations; endodontic treatments; periapical lesions; cysts; tumors; or history of trauma. Patients with lower third molars who had other radiographic signs of IAN injury, such as root darkening, canal diversion, and interruption of the canal’s white line, as well as those whose roots were not fully formed, were also excluded. Based on the literature review, the highest estimated prevalence of paresthesia is reported to be 20 % [3, 4]. We hypothesized that the IAN injury in the JAR^**+**^ group was twice that of the JAR^−^ group. As a result, with α = 0.05 and power = 80 %, we required 64 samples in both groups, but we selected 75 samples to increase the accuracy of the two groups. Using the random generating number method, 75 teeth with JAR and 75 teeth without JAR were chosen.

The CBCT images were acquired using a CBCT system (NewTom VGi, QR s.r.l., Italy) with a flat panel detector (FPD). The patients were positioned in the Frankfort plane parallel to the floor. The acquisition parameters were set as follows: current of 110 kVp; exposure time of 1.8 s; and field of view of 10 cm×5 cm. The field of view encompassed the mandibular third molars and the surrounding structures. A voxel size of 0.3 mm, with an interslice gap of 0.3 mm, was used to acquire images. The CBCT images were reviewed in NNT viewer version 8.0 under dimmed light. Image adjustments such as zoom, brightness, and contrast were also permitted.

**Fig. 1:**
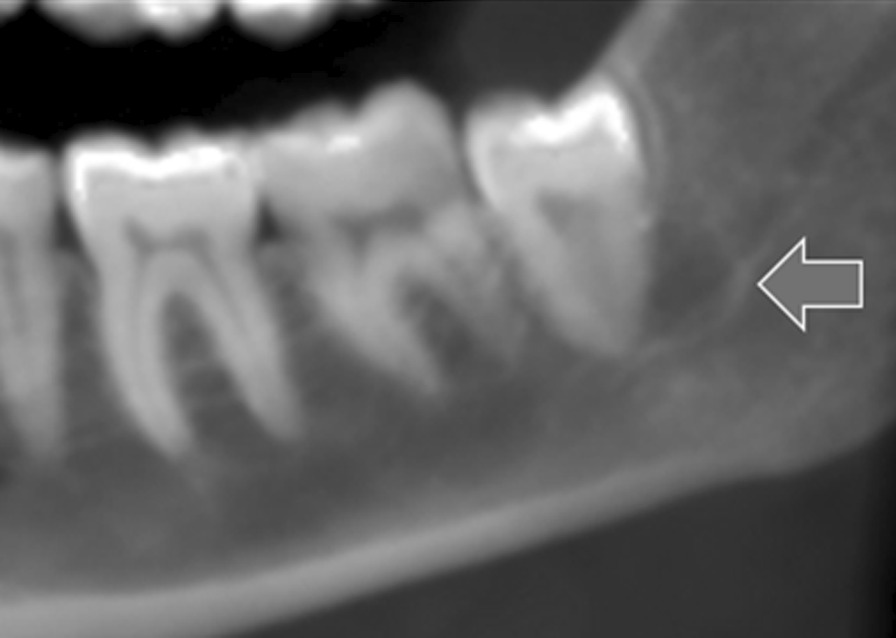
JAR associated with a lower third molar on reconstructed panoramic of CBCT images

The CBCT images were analyzed by two observers, one undergraduate student and one maxillofacial radiologist. The level of intra- and inter-examiner agreement was assessed. The mandibular third molars were evaluated in three aspects (sagittal, coronal, and axial) in CBCT images. The observers looked for JAR as a well-defined area of radiolucency apical or lateral to the roots of mandibular third molars. (Fig. 1).

**Fig. 2:**
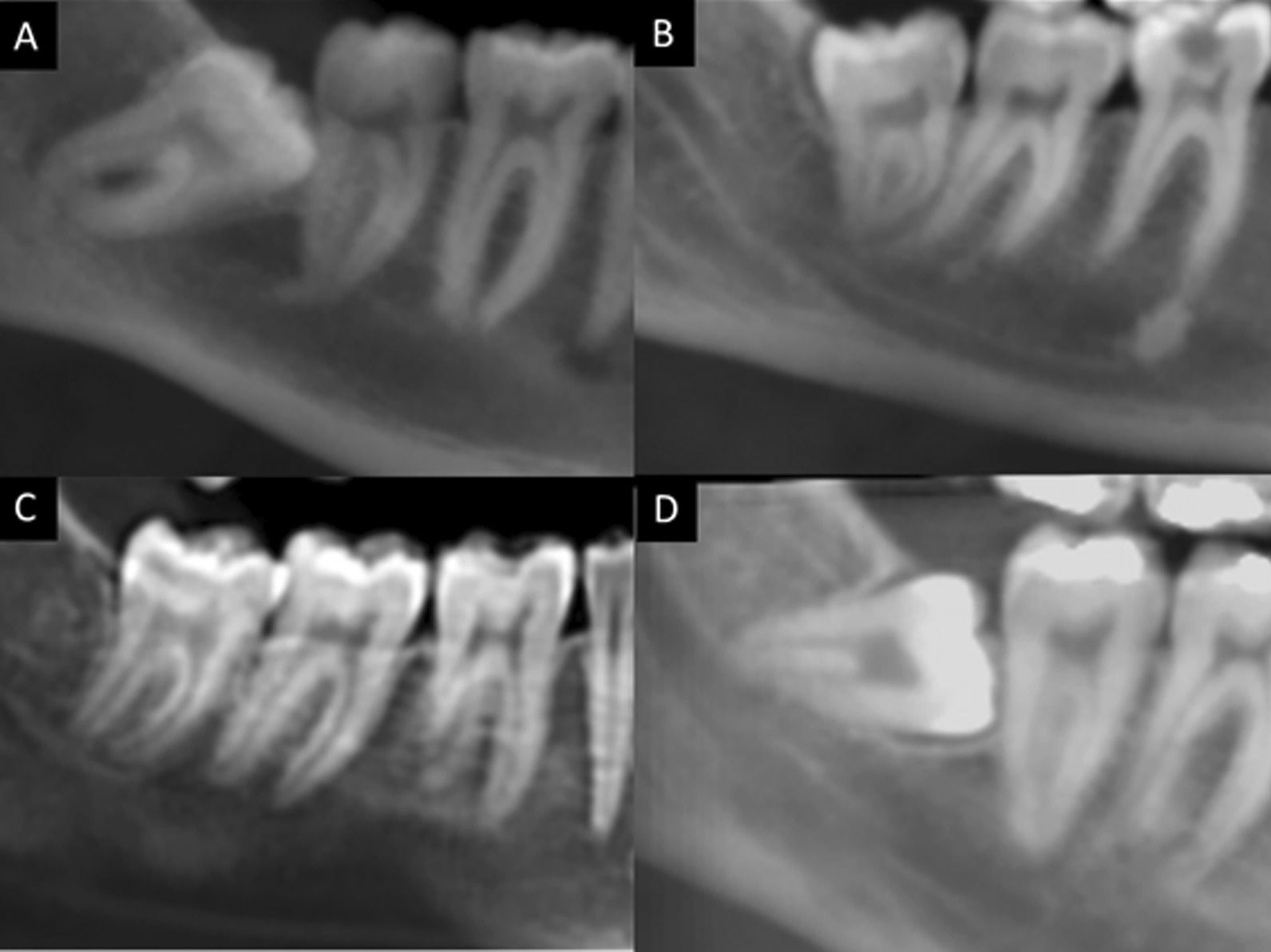
Classification of third molars based on angulation: mesioangular (**A**), distoangular (**B**), vertical (**C**), and horizontal (**D**)

According to Winter’s classification [16], the CBCT pictures were divided into four types of impaction: vertical, horizontal, mesioangular, and distoangular. (Fig. 2).

**Fig. 3:**
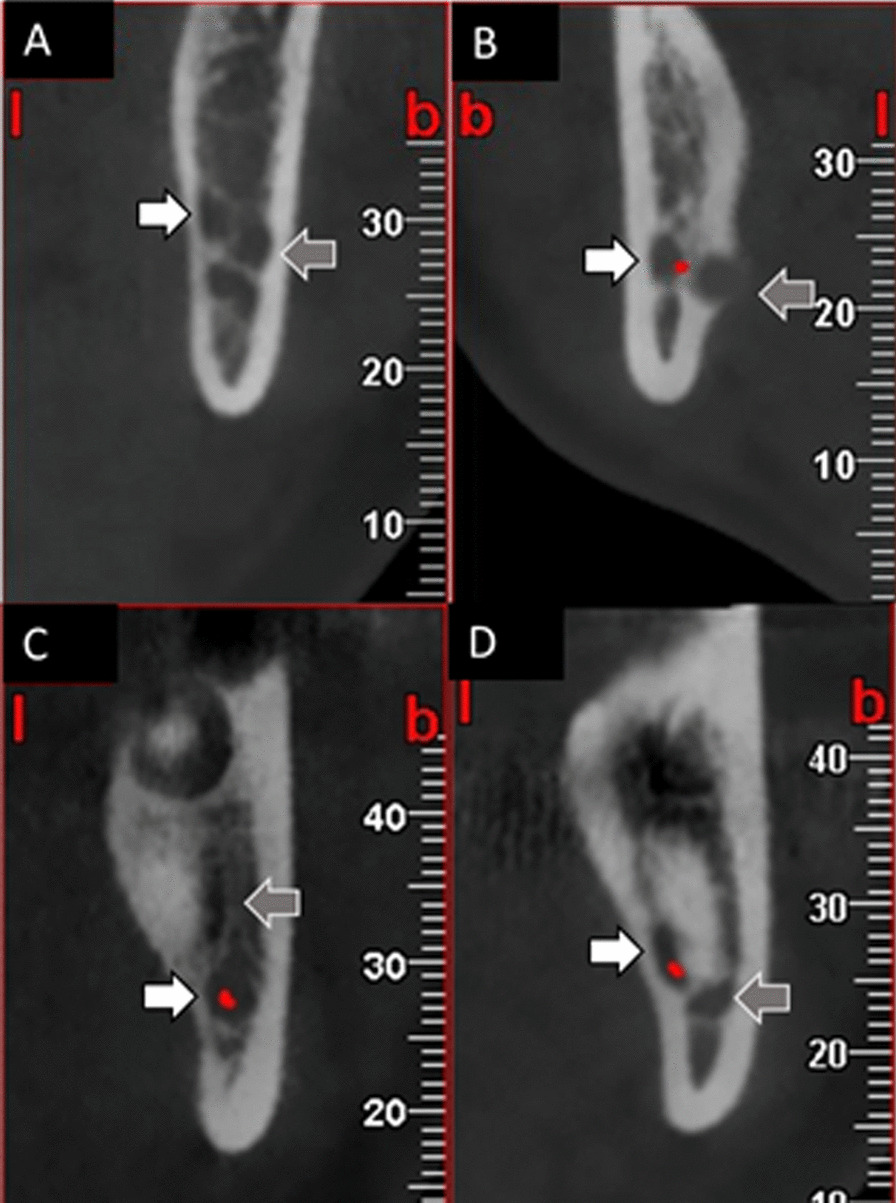
The position of JAR (gray arrows) relative to the mandibular canal (white arrows): buccal (**A**), lingual (**B**), superior (**C**), and inferior (**D**)

The position of JAR relative to the mandibular canal was categorized as buccal, lingual, superior, and inferior (Fig. 3).

**Fig. 4:**
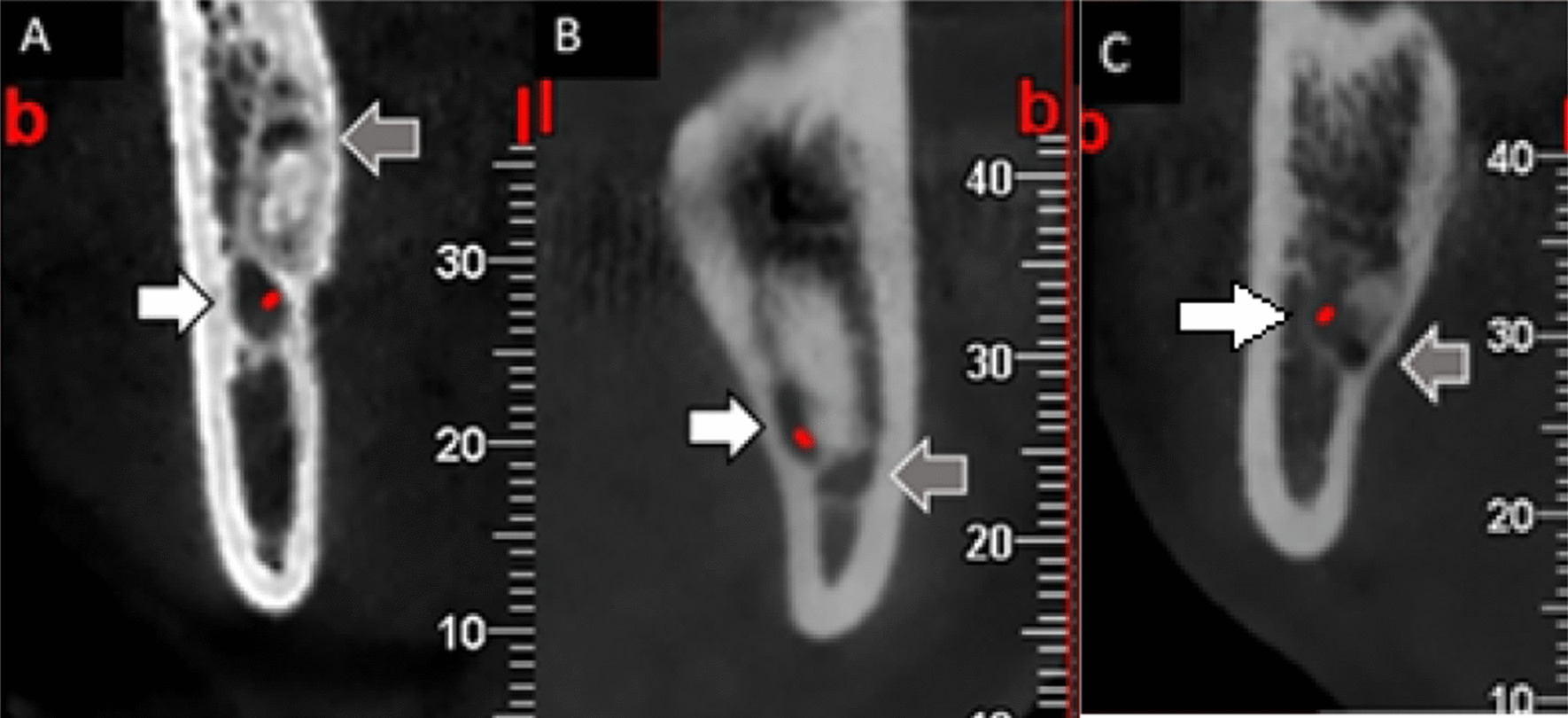
The relation of JAR (gray arrows) to IAN (white arrows): distant (**A**); in contact with IAN with preservation of the cortical plate (**B**); and in contact with IAN without preservation of the cortical plate (**C**)

In the JAR^−^ group, the location of the tooth apex relative to the mandibular canal was recorded. The proximity of JAR to the IAN canal was determined in three categories: (1) distant; (2) in contact with IAN with preservation of the cortical plate, and (3) in contact with IAN without preservation of the cortical plate (Fig. 4).

**Fig. 5:**
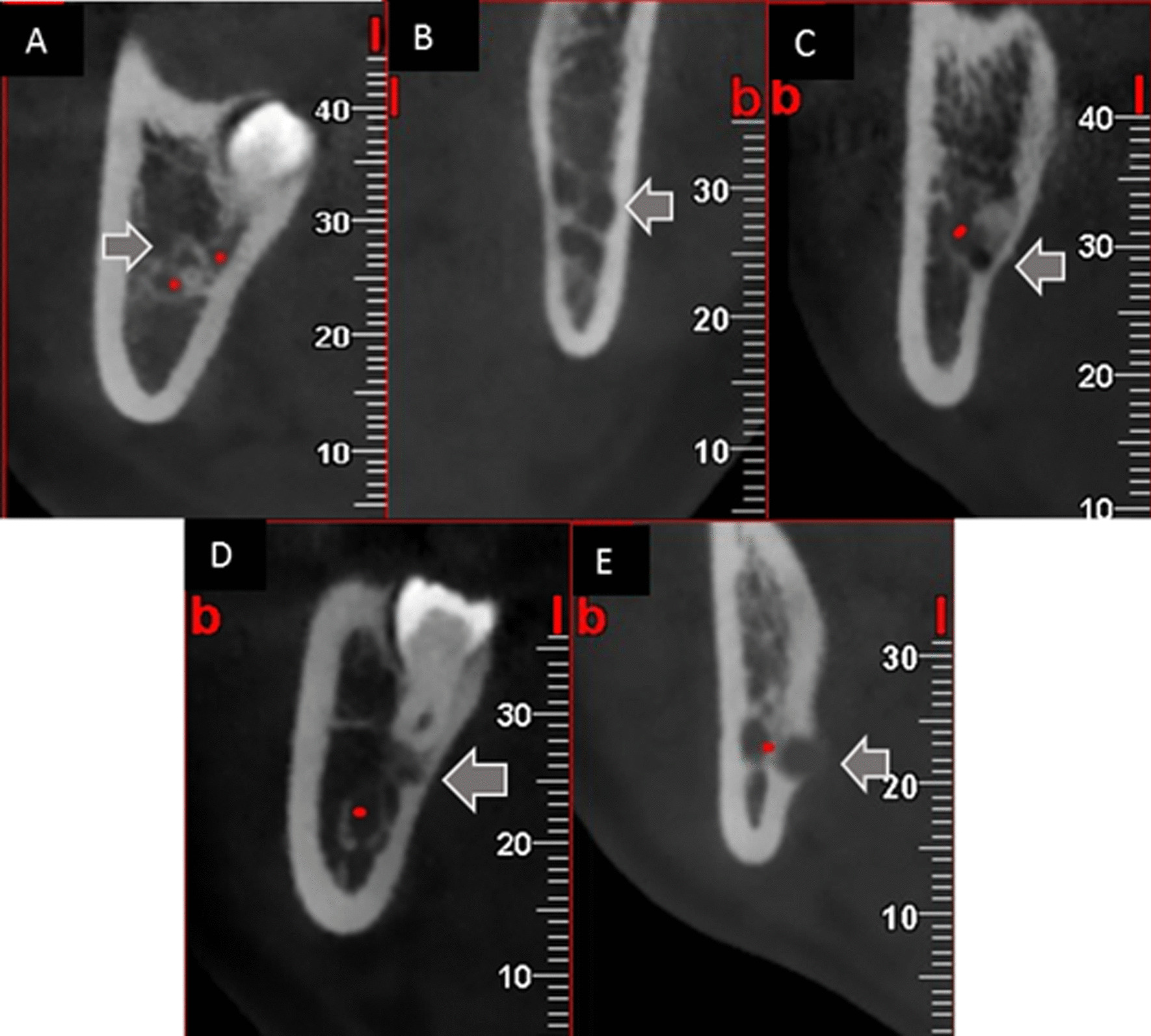
Lingual cortical plate thinning based on JAR (gray arrows) classification: J0 (**A**), J1 (**B**), J2 (**C**), J3 (**D**), and J4 (**E**)

The thinning of lingual cortical plates in the JAR region was recorded using the methodology proposed by Kapila et al. [17]. Briefly, when the remaining cortical thickness was three-fourths, half, or one-fourth of the maximal cortical thickness, the thinnest point of the cortical plate close to JAR was recorded and classified as J1, J2, and J3. The presence of cortical perforation was also recorded as J4, while an intact cortical plate was reported as J0 (Fig. 5). In the JAR^−^ group, the apex of teeth was considered rather than JAR.

Postoperative data were retrospectively retrieved from the clinical records. The JAR^+^ and JAR^−^ groups were asked about changes in feeling at three different time points: the initial days after surgery (temporary IAN injury), three and six months afterwards (permanent IAN injury). Based on Loescher et al. study [5], the patients were directly asked about any tingling sensation or numbness of the lips or chin to determine impairments in each examination period.

### Statistical analysis

Data were recorded in IBM SPSS for Windows version 26 (IBM Corp., Armonk, N.Y., USA). Chi-square and Fisher’s exact tests were performed for evaluating the association of JAR with IAN injury, JAR position, proximity to IAN, cortical plate thinning, and sex in the JAR^**+**^ and JAR^−^ groups. Independent samples *t*-test was also used for comparing the age of the JAR^**+**^ and JAR^−^ groups. The level of significance was set at *Р*<0.05. So, we initially performed univariate analysis to investigate the relationship between sex, age, lingual cortical thinning, tooth position according to IAN, proximity to IAN, and jar with paresthesia. Then, to control the effect of factors that were significant in univariate analysis, we entered items from univariate analysis with *P* < 0.15 in the logistic regression model.

## Results

There was an almost perfect intra-observer and inter-observer agreement (kappa coefficient = 0.9, 0.88 respectively). This study included 332 participants (545 mandibular third molars), all of whom were at least18 years old and had complete root formation. Overall, 75 (13.76 %) of 61 (18.37 %) individuals had JAR in their mandibular third molars. Only 14 out of 61 (22.95 %) patients had bilateral JAR. In contrast, 75 mandibular third molars from 56 patients, who did not have JAR, were chosen as the JAR^−^.

### Relationship between JAR and temporary IAN injury after surgery

Twenty-three cases of the JAR^**+**^ group (30.7 %) showed temporary IAN injury, whereas in the JAR^−^ group, only 12 cases showed temporary IAN injury after surgery. The prevalence of temporary IAN injury after surgery in the JAR^**+**^ group was significantly higher than the JAR^−^ group (*P* = 0.034). In the JAR^**+**^group, the risk of IAN injury was 2.32 times more likely than in the JAR^**−**^ group. The results of logistic regression analysis confirmed the relation between JAR and IAN injury (*P =* 0.036).

### Relationship between JAR and permanent IAN injury after surgery

All patients with IAN injury in the JAR^**+**^ and JAR^**−**^ groups recovered three and six months after surgery, and no permanent IAN injury was reported.

### Relationship between tooth angulation and temporary IAN injury

The tooth angulation in both groups with temporary IAN injury is shown in Table 1. Although the number of vertically angulated teeth with IAN injury in the JAR^**+**^ group was higher than the JAR^−^ group, there was no significant relationship between the tooth angulation and temporary IAN injury after surgery (*P* = 0.362).

**Table 1:** The IAN injury relation to anatomical structures and dental variables based on univariate analysis

Variables	JAR group	JAR- group	*P* value
*Sex no. (%)*			
Female	14 (73.7 %)	6 (60.0 %)	0.675
Male	5 (26.3 %)	4 (40.0 %)	
*Age mean ± SD*	26.05 ± 3.734	26.30 ± 4.001	0.870
*Tooth angulation N (%)*			
Mesioangular	9 (39.1 %)	7 (58.3 %)	0.362
Distoangular	2 (8.7 %)	1 (8.3 %)	
Vertical	8 (34.8 %)	1 (8.3 %)	
Horizontal	4 (17.4 %)	3 (25.0 %)	
*Proximity to IAN N (%)*			
Distant	3 (13.0 %)	0 (0.0 %)	0.305
Preserved cortex	6 (26.1 %)	4 (33.3 %)	
Without preserved cortex	14 (60.9 %)	8 (66.7 %)	
*Position N (%)*			
Superior	5 (21.7 %)	6 (50.0 %)	0.052
Inferior	3 (13.0 %)	2 (16.7 %)	
Buccal	4 (17.4 %)	2 (16.7 %)	
Lingual	11 (47.8 %)	2 (16.7 %)	
*Lingual cortical plate thinning N (%)*			
J0	1 (4.3 %)	5 (41.7 %)	0.012
J1	6 (26.1 %)	1 (8.3 %)	
J2	5 (21.7 %)	1 (8.3 %)	
J3	6 (26.1 %)	3 (25.0 %)	
J4	5 (21.7 %)	2 (16.7 %)	

### Relationship between proximity to IAN and temporary IAN injury

In the JAR^**+**^ group, 20 out of 23 teeth in contact with IAN showed temporary IAN injury, whereas 12 out of 12 teeth in contact with IAN, with or without cortical preservation, showed IAN injury in the JAR^−^ group. The proximity of teeth with temporary IAN injury to IAN is presented in Table 1. There was no significant relationship between proximity to IAN and temporary IAN injury (*P* = 0.305).

### Relationship between a position relative to IAN and temporary IAN injury

The majority of JAR teeth (11/23) showed IAN injury when they were lingual to IAN, whereas the majority of JAR^−^ teeth (6/12) with IAN injury were superior to the mandibular canal. There was an almost significant relationship between the JAR position and the presence of IAN injury (*P* = 0.052). Table 1 shows the position of teeth with IAN injury. According to the results of the regression analysis, there was no significant relationship between position and IAN injury.

### Relationship between lingual cortical plate thinning and temporary IAN injury

The majority of teeth with IAN injury in the JAR^+^ group (95.7 %) had lingual cortical plate thinning, whereas 58.3 % of teeth with IAN injury in the JAR^−^ group had lingual cortical plate thinning. There was a significant relationship between IAN injury and lingual cortical plate thinning (P = 0.012). The lingual cortical plate thinning in patients with IAN injury is shown in Table 1. As shown in Table 2, the relationship between lingual cortical thinning and IAN injury was disproved by regression analysis, The P-values for this relationship are shown in Table 2.

**Table 2:** The IAN injury relationships with other variables based on logistic regression analysis

Variables	*P *value	Odds ratio	95 % CI for odds ratio	
			Lower	Upper
Sex	0.719	1.189	0.463	3.049
Age	0.739	0.983	0.893	1.083
JAR	0.036	2.332	1.055	5.110
*Lingual cortical thinning*				
J1	0.318	1.917	0.535	6.872
J2	0.481	0.639	0.184	2.223
J3	0771	0.119	0.37	3.829
J4	0.269	2.064	0.571	7.462
*Position*				
Superior	0.535	1.488	0.424	5.215
Inferior	0.385	0.614	0.204	1.849
Buccal	0.724	1.182	0.468	2.984

## Discussion

In this study, we evaluated the presence of JAR and its relationship with the presence of IAN injury in CBCT images. Moreover, sex, age, thinning of the lingual cortical plate, position relative to IAN, and proximity to the mandibular canal were evaluated in the JAR^+^ and JAR^−^ groups. Three months after surgery, there was a significant association between JAR and the presence of temporary IAN injury (*P* = 0.034), whereas no cases of permanent IAN injury were detected in either of the groups. According to univariate analysis, only the relationship between JAR, lingual cortical thinning with IAN injury were significant. Position according to IAN was almost significant. Logistic regression analysis revealed that the lingual cortical thinning and position according to IAN were not significant. Only the relation between JAR and IAN injury was found to be statistically significant. As a result, JAR was the main factor that affected IAN injury.

Seven radiological signs are considered to be indicative of a close relationship between the impacted mandibular third molars and the inferior alveolar canal. Only three of these signs seem to be significantly related to the inferior alveolar nerve injury, including the canal diversion, darkening of the root, and interruption in the white line of IAN [8]. Moreover, JAR and deviation of the canal were significantly associated with nerve injury [4]. In the present study, the main risk factor for injury was the presence of JAR in CBCT images. 18.37 % of the patients had JAR signs in CBCT images. In two studies by Nascimento et al. [12, 14], 15.9 and 32.6 % of patients showed JAR in CBCT images. In addition, Yalcin and Artas reported that 33 % of patients showed JAR in CBCT images. In our study, the number of female patients in the JAR group was substantially greater than in the JAR^**−**^group (*P* = 0.037). According to Nascimento et al., the probability of JAR identification in female patients is almost twice that of male patients [13].

Recently, it has been hypothesized that JAR is the initial area of focal osseous dysplasia [14]. In this regard, Umar et al. [10] reported that JAR originates from the superimposition of a large cancellous bone on the mandibular canal. Similarly, Gilvetti et al. [9] studied 50 cases of JAR in panoramic images and found no temporary or permanent IAN injury after at least 18 months. Yalcin and Artas [15] confirmed this result, as they found no significant relationship between JAR and the mandibular canal. In a study by Nascimento et al., in most JAR cases, the mandibular canal contacts the JAR [12]. The present study found that most of the patients with IAN injury had JAR. Logistic regression analysis confirmed the results of the univariate analysis that a significant relationship exists between JAR and IAN injury (*P =* 0.036).

In the present study, the majority of JARs (90.7 %) were in contact with IAN, with or without cortical border preservation. This finding is in line with the results reported by Nascimento et al., which revealed that only 6.4 % of cases were located distant from the mandibular canal, while most cases were in contact with the canal [12]. In contrast, Kapila et al. showed that 28.57 % of JAR cases were in contact with the mandibular canal in CBCT images [18]. Similarly, Yalcin and Artas found that JAR was mostly (76.3 %) distant from the mandibular canal [15]. Based on our finding, 32 % of JAR^+^, who had contact with IAN, suffered from IAN injury.

Overall, determining the position of the JAR is important, considering the possible need for special care during surgery [12]. In this regard, Ghaeminia reported that during surgery, the lingual side of the mandibular canal in the third molar region is more vulnerable to unfavorable forces [19]. Various outcomes have been reported in the literature for the JAR position. Nascimento et al. found that JAR was in the lingual position relative to IAN (59.6 %) [12]. However, in a study by Kapila et al., the most frequent positions were buccal and superior to IAN [17]. Furthermore, Yalcin and Artas showed that JAR was mostly in the superior position [15]. In our study, lingual and buccal positions were the most common positions. Despite the fact that the majority of JARs with IAN injury were on the lingual side of the mandibular canal; logistic regression analysis disproved the relationship between position and IAN injury.

Moreover, Yalcin and Artas found cortical thinning in 67 % of the cases in their study. Besides, Kapila et al. reported that cortical plates thinning were significantly more common in JAR^**+**^ group than JAR^−^ group (70 % vs. 37 %). They postulated that cortical plate thinning could be responsible for postoperative IAN injury following the extraction of third molars. In the present study, 84 % of JAR cases showed at least some degree of lingual cortical plate thinning, and even 16 % of cases were perforated. However, considering univariate analysis, we found a significant relationship between the presence of JAR and thinning of the cortical plate (*P* = 0.012), but by modulating the effects of variables in logistic regression analysis, this relationship disappeared.

In the current study, most cases of JAR (54.7 %) were detected in teeth with a mesioangular position; however, there was no significant difference between the groups (*P* = 0.346). In this regard, Kapila et al. found the mesioangular position to be the most common one [18]. However, they did not include a control group to analyze the significance of their findings. On the other hand, in the study by Nascimento et al., although panoramic images were examined, JAR was associated with vertically positioned teeth [13]. Yalcin and Artas also found that the vertical position was the most common angulation related to JAR in CBCT images; however, they did not include a control group in their study [15]. The majority of IAN injury is found in teeth with the mesioangular position. (25 % JAR^**+**^ vs. 20 % JAR^−^ groups)

Loescher et al. postulated that the patient’s subjective report is the most sensitive indicator of abnormal sensation and that tests cannot detect minor sensory disturbances [5]. Therefore, we did not use any quantitative tests in our study and identified the patients qualitatively by asking them about any tingling sensation or numbness of the lips or the chin. In their study, the patient follow-up was conducted via phone calls, which can increase the number of participants in the research while also being more cost-effective [20].

Overall, the current study revealed that temporary IAN injury is significantly more common in patients with JAR^**+**^ (on CBCT images) than the JAR^−^. However, no permanent IAN injury was detected during the three-month follow-up. A study by Alling et al. found that 96 % of inferior alveolar nerve lesions recovered within 4–8 weeks following surgery, which is consistent with our findings [21]. Only one other study investigated the relationship between IAN injury and JAR in panoramic images, which found no significant relationship [9]. Since JAR detection is more accurate in CBCT images than in panoramic images, CBCT images were used in this study [14].

## Conclusions

JAR was significantly associated with temporary IAN injury. Also, JAR was also shown to be significantly more common in female patients than in male ones. IAN injury showed no significant relationship with the tooth angulation, position to IAN and proximity to the mandibular canal, lingual cortical plate thinning, sex, and age In the present study, Postoperative data were retrospectively retrieved from the clinical records. It is suggested that clinical examination will be used to determine the exact type of IAN injury. Further research with a larger sample size is required to confirm the association between JAR and IAN injury.

## Data Availability

The datasets used and analyzed during the current study are available from the corresponding author.
